# Intensive versus standard statin therapy in acute ischemic stroke: a comparative study on the risk of pneumonia and multidrug-resistant bacterial infections

**DOI:** 10.3389/fneur.2026.1801279

**Published:** 2026-07-15

**Authors:** Zhijun Wen, Zhenming Yang, Yirui Huang, Jianhua Cheng

**Affiliations:** 1 Department of Neurology, The First Affiliated Hospital of Wenzhou Medical University, Wenzhou, Zhejiang, China; 2 Department of Clinical Laboratory, Wenzhou Hospital of Integrated Traditional Chinese and Western Medicine, Wenzhou, Zhejiang, China

**Keywords:** atorvastatin, intensive treatment, ischemic stroke, post-stroke pneumonia, risk factors

## Abstract

**Background:**

The use of intensive statin therapy in acute ischemic stroke (AIS) is common; however, its association with post-stroke pneumonia (PSP) remains uncertain. This study aimed to determine whether intensive-dose atorvastatin (≥40 mg/d) increases the risk, severity, or incidence of multidrug-resistant (MDR) bacterial infections in PSP compared to standard-dose therapy (20 mg/d).

**Methods:**

We retrospectively analyzed 4,843 AIS patients admitted to the First Affiliated Hospital of Wenzhou Medical University between January 2020 and January 2022. After applying inclusion and exclusion criteria, 3,199 patients were included. Propensity score matching (PSM) was used to balance baseline characteristics between the intensive and standard atorvastatin groups. The incidence of PSP, pneumonia severity (evaluated by CURB-65 scores), and MDR bacterial infection rates were compared. Multivariate logistic regression was applied to identify independent risk factors for PSP.

**Results:**

After PSM, no significant difference was observed in PSP incidence between the intensive and standard atorvastatin groups (8.8% vs. 8.3%, *p* = 0.691). Multivariate regression confirmed that intensive treatment was not associated with increased PSP risk, either before (OR = 1.074, 95% CI: 0.756–1.525) or after matching (OR = 1.002, 95% CI: 0.672–1.492). Among the 173 PSP patients, there were no significant differences in the proportion of severe pneumonia (21.4% vs. 20.2%, *p* = 0.863) or MDR bacterial infection incidence (9.5% vs. 10.1%, *p* = 0.897) between the two groups.

**Conclusion:**

High-intensity atorvastatin therapy during the acute phase of ischemic stroke did not increase the risk or severity of pneumonia, nor did it elevate MDR bacterial infections. These findings suggest that intensive atorvastatin therapy is not associated with an increased risk of PSP and offer insights for optimizing clinical treatment strategies.

## 1 Introduction

Stroke as the leading cause of death in China, is often complicated by post-stroke infections, particularly post-stroke pneumonia (PSP) (1), which can lead to clinical deterioration and increased mortality (2–4). PSP not only significantly prolongs hospital stays but also elevates the risk of patient death. The prevention and management of PSP have become crucial for improving stroke outcomes. Current clinical attempts, such as prophylactic antibiotic therapy (5) and *β*-blocker intervention (6), have failed to effectively reduce the incidence of PSP. Based on the “stroke-induced immunodepression” theory, immunomodulation may emerge as a novel preventive and therapeutic direction (7).

Statins are cornerstone drugs in the early treatment of acute ischemic stroke. Beyond their lipid-lowering effects, statins possess anti-inflammatory, immunomodulatory, antioxidant, and endothelial-stabilizing properties (8, 9). However, the relationship between these pleiotropic effects and the risk of post-stroke pneumonia remains controversial. Existing research findings can be broadly categorized into three groups: (1) Increased risk of PSP (10): Becker et al. found that early statin use (within 3 days) after ischemic stroke might be associated with an increased risk of infection within the subsequent 15 days (OR = 7.21). (2) No effect on PSP (11, 12): Rodríguez et al. found that prior statin use did not seem to affect the frequency of in-hospital infections in ischemic stroke patients, and Li et al. found no additional benefit in reducing PSP for AIS patients using statins in the acute phase compared to non-users. (3) Reduced risk of PSP (13–15): Scheitz et al. found that continued statin use (compared to non-use) in patients with acute ischemic stroke receiving thrombolytic therapy might be associated with a reduced risk of PSP, although its impact on long-term functional outcomes and mortality was not significant. In-depth analysis suggests that differences in the type and dosage of statins used may be key underlying factors contributing to these inconsistent conclusions. For instance, a study on the impact of different statin doses on sepsis outcomes found that the beneficial effect of pre-administrated statins on sepsis outcomes significantly increased with higher doses (16). Another study found that statin therapy in the acute phase of ischemic stroke did not appear to increase the risk or severity of PSP. The authors of that study further suggested that the lack of consideration for specific statin types and dosages might explain this finding (12).

Current guidelines for the early treatment of acute ischemic stroke recommend routine intensive statin therapy for lipid lowering (17). However, against the backdrop of the increasing prevalence of intensive statin therapy, given the pleiotropic effects of statins on acute ischemic stroke and the aforementioned research controversies, coupled with the fact that most previous studies have focused on the impact of statin use versus non-use on post-stroke pneumonia, there remains a lack of systematic investigation into the specific effects of particular statin doses—especially intensive doses—on the risk of post-stroke pneumonia. Furthermore, no studies have examined whether intensive statin therapy during the acute phase influences the etiological distribution of causative pathogens or the prevalence of drug-resistant bacteria in post-stroke pneumonia. Moreover, a substantial portion of the significant discrepancies in prior findings regarding the association between statins and post-stroke pneumonia risk may be attributable to study design limitations and inadequate control of confounding factors. For instance, in observational studies, patients receiving intensive statin therapy tend to have better baseline characteristics (e.g., younger age, less severe neurological deficits, and fewer comorbidities), which are inherently protective factors against post-stroke pneumonia. If not properly controlled, such biases can easily generate spurious associations suggesting a protective effect of intensive statin therapy. Propensity score matching is an effective method for addressing such selection bias, as it creates a virtually randomized controlled environment with balanced baseline characteristics, enabling a more accurate estimation of the true treatment effects. Therefore, this study employed propensity score matching to control for confounding variables and investigated the effects of different doses of atorvastatin on post-stroke pneumonia, aiming to clarify the impact of high-intensity atorvastatin on the risk of post-stroke pneumonia and to provide evidence-based support for clinical management.

## 2 Materials and methods

### 2.1 Study population

We retrospectively collected clinical data of patients with Acute Ischemic Stroke (AIS) admitted to the First Affiliated Hospital of Wenzhou Medical University between January 2020 and January 2022 from the electronic medical record system. Patients who met the following inclusion criteria were enrolled: (1) diagnosis of AIS according to the criteria established by the Chinese Society of Neurology (18); (2) Age ≥ 18 years; (3) Time from onset to admission ≤ 3 days. The exclusion criteria were as follows: (1) History of prior stroke or transient ischemic attack (TIA); (2) Prophylactic antibiotic treatment administered pre-stroke or during the stroke hospitalization; (3) Pre-existing infectious, autoimmune diseases, or any malignancies; (4) Pre-stroke statin medication use; (5) Pregnancy; (6) Infections during the stroke hospitalization other than PSP; (7) Severe hepatic or renal dysfunction; (8) Incomplete clinical data. For this study, the completeness of the original data was verified. Data with missing values for key variables, including post-stroke pneumonia outcomes, atorvastatin dosage, and major covariates, were excluded. Given the low proportion of missing data for each variable (<5%), no methods such as multiple imputation were applied; instead, a complete-case analysis was adopted for statistical analysis.

### 2.2 Ethical considerations

This study was approved by the Ethics Committee of the First Affiliated Hospital of Wenzhou Medical University (Ethics Approval Number: KY2022-R008) and was conducted in accordance with the principles of the Declaration of Helsinki. As a retrospective observational study that met the following conditions: (1) all data were derived from de-identified archival information from the hospital’s electronic medical record system; (2) the research process involved no additional interventions; (3) data analysis was performed on a fully anonymized dataset (with sensitive information such as names and ID numbers removed); and (4) the study results would not disclose individual patient treatment details, the Ethics Committee granted a waiver of informed consent in compliance with the Declaration of Helsinki and relevant Chinese regulations (“Ethical Review Measures for Biomedical Research Involving Humans”). The entire study adhered to the “Management Specifications for Privacy and Data Sharing in Medical Research (2021 Edition).”

### 2.3 Data collection

Baseline characteristics were collected from electronic medical records, including:

- Demographic parameters: age and gender.
- Clinical characteristics: systolic blood pressure, diastolic blood pressure, respiratory rate, presence of nasogastric tube, endotracheal intubation or tracheostomy, dysphagia, admission NIHSS score, and length of hospital stay.
- Medical history: smoking history, alcohol consumption history, hypertension, diabetes mellitus, coronary heart disease, atrial fibrillation, and chronic obstructive pulmonary disease (COPD).
- Laboratory parameters: white blood cell count, lymphocyte count, serum albumin level, total cholesterol (TC), triglycerides (TG), high-density lipoprotein cholesterol (HDL-C), low-density lipoprotein cholesterol (LDL-C), and blood Urea nitrogen.
- Imaging characteristics: stroke location (anterior circulation, both anterior and posterior circulation, and posterior circulation).
- Others: Trial of Org 10,172 in acute stroke treatment (TOAST) classification.

### 2.4 Definition of high-intensity atorvastatin therapy

Based on a previous study (19) and the American Heart Association’s definition for High-intensity atorvastatin therapy (20), intensive treatment in this study was defined as the administration of atorvastatin at a dose ≥40 mg/d, initiated within 72 h of symptom onset (acute phase). The standard treatment was defined as 20 mg/d.

### 2.5 Diagnostic criteria for post-stroke pneumonia

PSP was diagnosed based on the presence of new or progressive infiltrative changes on chest imaging after stroke onset, plus at least two of the following clinical symptoms/signs (21):

1. New or worsened cough/sputum production, with or without chest pain;
2. Signs of pulmonary consolidation and/or moist rales;
3. Fever (body temperature ≥38 °C);
4. White blood cell count ≥10 × 10^9^/L or ≤4 × 10^9^/L, with or without a left shift.

Conditions with similar clinical presentations, such as lung cancer, pulmonary tuberculosis, pulmonary edema, atelectasis, non-infectious interstitial lung disease, and pulmonary embolism, were rigorously excluded.

### 2.6 Severity of post-stroke pneumonia

The severity of PSP was assessed using the CURB-65 score during hospitalization (22). A CURB-65 score ≥3 was defined as severe pneumonia. The CURB-65 score is calculated as follows (1 point for each):

1. Confusion;
2. Blood Urea nitrogen >7 mmol/L;
3. Respiratory rate ≥30 breaths/min;
4. Systolic blood pressure <90 mmHg or Diastolic blood pressure ≤60 mmHg;
5. Age ≥65 years.

### 2.7 Definition of multidrug-resistant organisms

According to a currently internationally authoritative and widely cited definitive publication (23), multidrug-resistant organisms (MDROs) in this study were defined as bacteria acquiring resistance to three or more classes of antimicrobial agents listed in the antimicrobial spectrum classification.

### 2.8 Statistical analysis

Statistical analyses were performed using IBM SPSS Statistics for Windows, version 26.0 (IBM Corp., Armonk, NY, United States), and graph generation was conducted using RStudio for Windows, version 2026.01.1 Build 403 (^©^2009–2026 Posit Software, PBC). The Kolmogorov–Smirnov test was used to determine whether the included variables followed a normal distribution. Normally distributed continuous variables were compared using Student’s t-test and one-way analysis of variance, and results were expressed as mean ± standard deviation (mean ± SD). For non-normally distributed continuous variables, the Mann–Whitney U test was used to assess differences between two groups, and data were presented as median with interquartile range (IQR). Categorical variables were analyzed using the chi-square (χ^2^) test or Fisher’s exact test, and expressed as numbers (*n*) and percentages (%). The significance level was set at two-tailed *p* < 0.05. Logistic regression analysis was employed to investigate the association between the development of post-stroke pneumonia and high-intensity atorvastatin therapy during the acute phase. Multivariable logistic regression analysis included all variables with *p* < 0.05 in the univariable logistic regression analysis and covariates associated with post-stroke pneumonia in previous literature. Logistic regression models were analyzed using both the enter method and the conditional method, before and after propensity score matching, respectively. All tests were two-sided, and a *p* < 0.05 was considered statistically significant.

### 2.9 Propensity score matching (PSM)

The fundamental principle of Propensity Score Matching (PSM) is to calculate the probability of each patient receiving intensive therapy (i.e., the propensity score) and then match patients from the intensive therapy group with those from the standard therapy group who have similar scores, thereby constructing comparable cohorts with balanced baseline characteristics across different dose groups. In this study, propensity scores were obtained through logistic regression analysis, with acute-phase high-intensity atorvastatin therapy as the dependent variable. Based on the following considerations: (1) 1:1 matching minimizes matching error and improves baseline balance between groups; (2) the sample size of the standard therapy group in this study was relatively small, and adopting 1:2 matching could potentially compromise matching quality; (3) this study prioritized controlling confounding bias over maximizing sample size, thus the 1:1 matching approach, which offers higher matching quality, was selected. Patients in the high-intensity atorvastatin therapy group were matched with those in the standard atorvastatin therapy group using a caliper value of 0.02. To maximize control over confounding bias and ensure model accuracy, based on previously reported risk factors for post-stroke pneumonia (PSP) in the literature and variables that differed significantly between groups in the univariate analysis (*p* < 0.05), the following covariates were pre-selected and included for propensity score matching: age, sex, smoking history, drinking history, history of hypertension, history of atrial fibrillation, history of coronary heart disease, impaired consciousness, indwelling nasogastric tube, endotracheal intubation/tracheostomy, stroke location, TOAST classification, lymphocyte count, albumin, triglycerides, low-density lipoprotein, and high-density lipoprotein. The effectiveness of matching was assessed using the Standardized Mean Difference (SMD). The SMD is not influenced by sample size and provides a more objective reflection of between-group balance. Generally, an SMD < 0.1 indicates well-controlled differences between groups (24). If the SMD for all covariates is less than 0.1 after matching, the matching is considered successful, indicating that the two groups are balanced with respect to known confounding factors. After matching, *p* values were no longer used to assess balance to avoid the influence of sample size on statistical significance.

## 3 Results

### 3.1 Baseline characteristics

**Figure 1:**
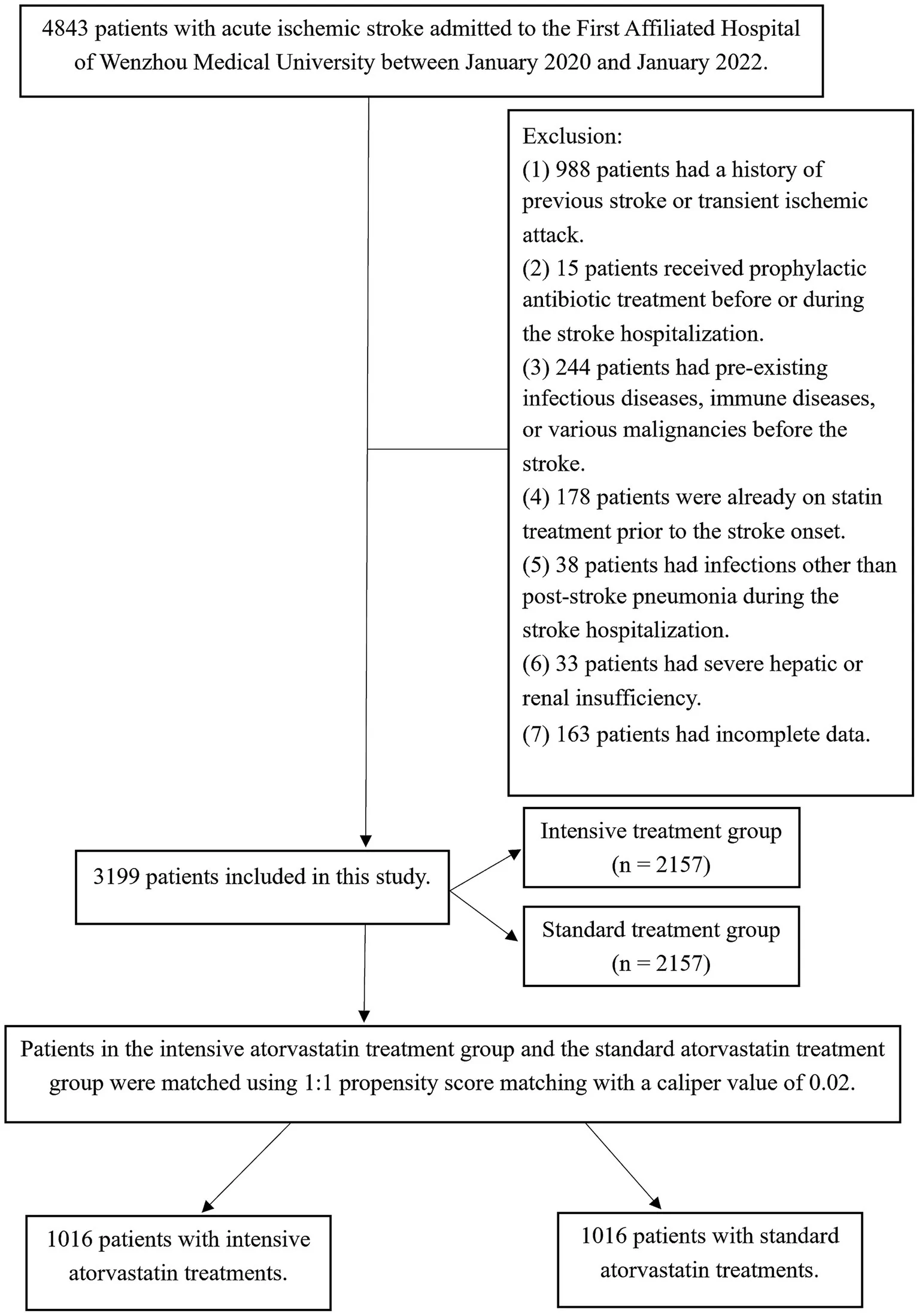
Flowchart of study participant enrollment.

During the study period, a total of 4,843 patients with acute ischemic stroke who were admitted to and treated in the Department of Neurology at the First Affiliated Hospital of Wenzhou Medical University between January 2020 and January 2022 were initially identified (Figure 1). After applying the inclusion and exclusion criteria, 3,199 patients were ultimately included in the analysis. In all patients, 2,101 were males (65.7%), with a mean age of 68.00 (58.00, 76.00) years. A total of 2,157 (67.4%) patients received High-intensity atorvastatin therapy in the acute phase, and 1,042 (32.6%) patients received standard atorvastatin treatment in the acute phase. Among the included cohort of 3,199 patients, 243 (7.6%) patients developed pneumonia after acute ischemic stroke.

### 3.2 Baseline characteristics of AIS patients in the intensive and standard atorvastatin treatment groups before and after propensity score matching

**Figure 2:**
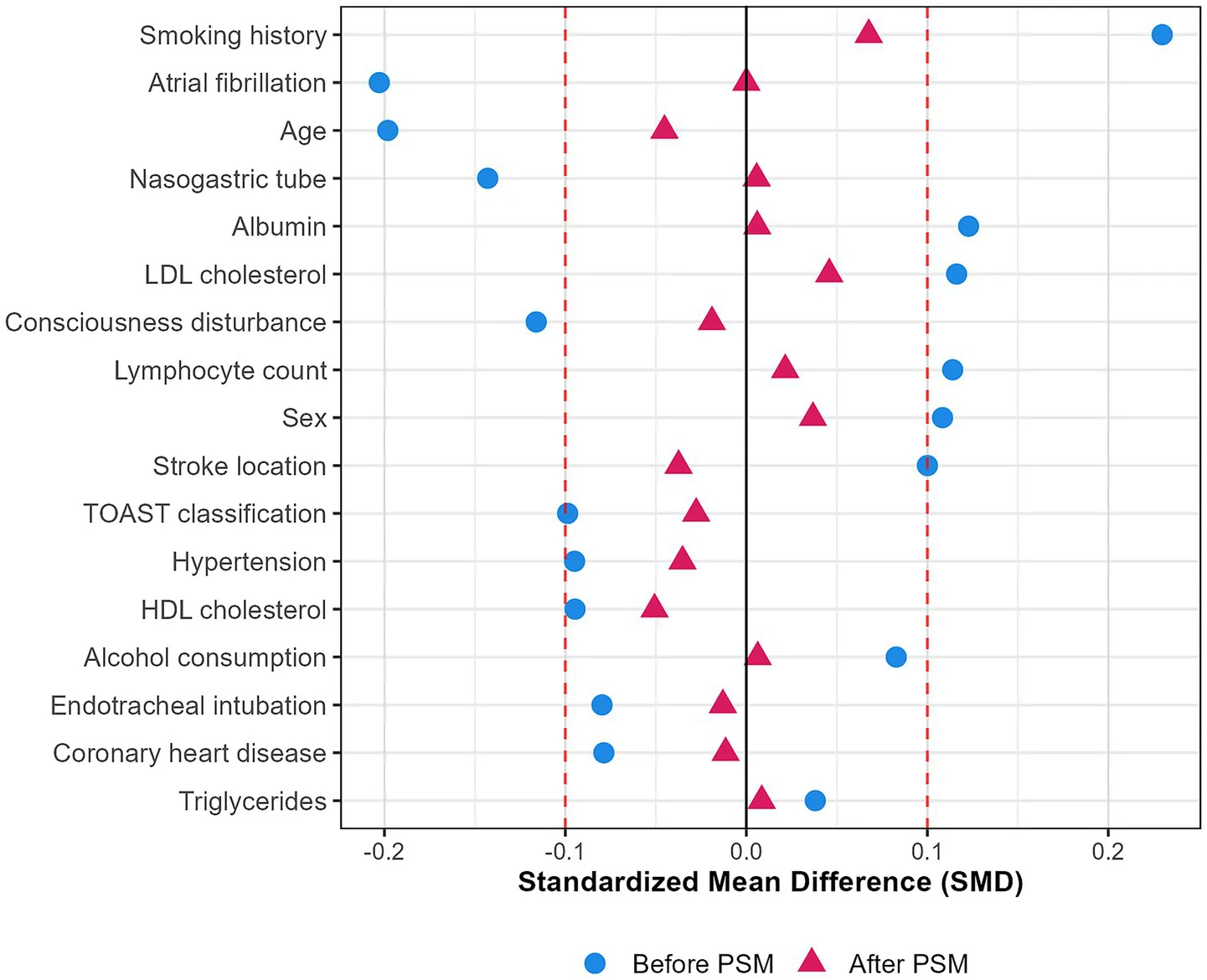
Standardized mean differences of each variable before and after propensity score matching (PSM).

To evaluate the impact of high-intensity atorvastatin therapy on the risk of post-stroke pneumonia in patients with acute ischemic stroke, this study employed propensity score matching (PSM) to control for potential confounding bias. As visually presented in Figure 2, the standardized mean differences (SMD) of each covariate between the high-intensity atorvastatin therapy group and the standard therapy group in patients with acute ischemic stroke are displayed before and after PSM. Blue dots represent the between-group differences before matching, while red triangles represent those after matching. A smaller SMD indicates better balance of the variable between the two groups. Generally, an SMD of < 0.1 is considered to indicate well-controlled between-group differences. After matching, the SMD for variables was less than 0.1, indicating that the baseline covariates of the two groups achieved good balance following PSM (although several variables remained statistically different due to sample size effects, all SMDs were <0.1, indicating acceptable covariate balance), thereby providing a comparable foundation for subsequent comparisons of post-stroke pneumonia outcomes between the two groups.

The baseline characteristics of the intensive and standard atorvastatin treatment groups, both before and after propensity score matching (PSM), are summarized in Table 1. Before matching, significant differences were observed in multiple demographic, clinical, and laboratory parameters. Following 1:1 PSM, 2,032 patients (1,016 pairs) were successfully matched, and all baseline characteristics were well-balanced between the two groups, confirming the effectiveness of the matching process. As detailed in Table 1, prior to PSM, patients in the intensive atorvastatin group were younger, had a higher proportion of males, smokers, and drinkers, and exhibited a different comorbidity and intervention profile compared to the standard-dose group. These pre-existing imbalances were effectively mitigated after PSM, creating comparable groups for a robust evaluation of the treatment effect on the outcomes of interest.

**Table 1:** Baseline characteristics of AIS patients in the intensive and standard atorvastatin treatment groups before and after propensity score matching.

Variable	Before PSM			After PSM		
	Intensive treatment (*n* = 2057)	Standard treatment (*n* = 1,042)	*p* value	Intensive treatment (*n* = 1,016)	Standard treatment (*n* = 1,016)	*p* value
Age, years, median (IQR)	67 (58,75)	70 (60,79)	***<0.001***	69 (60,70)	70 (59,78)	0.217
Male, *n* (%)	1,453 (67.4)	648 (62.2)	***0.004***	655 (64.5)	637 (62.7)	0.407
Smoking history, *n* (%)	968 (44.9)	351 (33.7)	***<0.001***	384 (37.8)	351 (34.5)	0.128
Alcohol history, *n* (%)	776 (36.0)	334 (32.1)	***0.029***	334 (32.9)	331 (32.6)	0.887
Medical history, *n* (%)						
Hypertension	1,345 (62.4)	697 (66.9)	***0.012***	660 (65.0)	677 (66.6)	0.427
Diabetes mellitus	577 (26.8)	257 (24.7)	0.208	272 (26.8)	249 (24.5)	0.243
Atrial fibrillation	94 (4.4)	98 (9.4)	***<0.001***	76 (7.5)	76 (7.5)	1.000
Coronary heart disease	45 (2.1)	35 (3.4)	***0.031***	30 (3.0)	32 (3.1)	0.796
COPD	13 (0.6)	7 (0.7)	0.816	8 (0.8)	6 (0.6)	0.592
Clinical features, *n* (%)						
Consciousness disorder	176 (8.2)	121 (11.6)	***0.002***	107 (10.5)	113 (11.1)	0.668
Nasogastric tube	214 (9.9)	152 (14.6)	***<0.001***	141 (13.9)	139 (13.7)	0.898
Endotracheal intubation/tracheostomy	35 (1.6)	29 (2.8)	***0.028***	23 (2.3)	25 (2.5)	0.770
Dysphagia	241 (11.2)	120 (11.5)	0.774	119 (11.7)	115 (11.3)	0.781
Admission NIHSS score, median (IQR)	3 (1,6)	3 (1,7)	0.223	3 (1,7)	3 (1,7)	***0.005***
Length of hospital stay, days, median (IQR)	8 (7,11)	8 (7,11)	0.916	8 (7,11)	8 (7,11)	0.372
Post-stroke pneumonia, *n* (%)	144 (6.7)	99 (9.5)	***0.005***	89 (8.8)	84 (8.3)	0.691
Stroke location, *n* (%)						
Anterior circulation	1,301 (60.3)	631 (60.6)	***<0.001***	650 (64.0)	612 (60.2)	***0.013***
Anterior & posterior circulation	565 (26.2)	320 (30.7)		256 (25.2)	313 (30.8)	
Posterior circulation	291 (13.5)	91 (8.7)		110 (10.8)	91 (9.0)	
TOAST classification, *n* (%)						
LAA	1,667 (77.3)	740 (71.0)	***<0.001***	752 (74.0)	731 (71.9)	0.585
CE	244 (11.3)	180 (17.3)		149 (14.7)	164 (16.1)	
SVO	161 (7.5)	91 (8.7)		78 (7.7)	90 (8.9)	
ODE	20 (0.9)	7 (0.7)		6 (0.6)	7 (0.7)	
UDE	65 (3.0)	24 (2.3)		31 (3.1)	24 (2.4)	
Laboratory parameters, median (IQR)						
White blood cell count (x10^9^/L)	7.02 (5.81,8.52)	7.14 (5.79,8.54)	0.597	6.98 (5.80,8.43)	7.09 (5.78,8.51)	0.521
Lymphocyte count (x10^9^/L)	1.69 (1.33,2.11)	1.6 (1.24,2.06)	***0.001***	1.62 (1.28,2.05)	1.61 (1.24,2.07)	0.557
Albumin (g/L)	37.9 (35.6,40.2)	37.1 (35.0,39.8)	***<0.001***	37.4 (35.3,39.9)	37.2 (35.1,39.9)	0.430
Total cholesterol (mmol/L)	4.51 (3.75,5.31)	4.40 (3.65,5.27)	0.079	4.45 (3.73,5.25)	4.42 (3.67,5.27)	0.645
Triglycerides (mmol/L)	1.44 (1.07,2.00)	1.34 (0.99,1.91)	***0.003***	1.39 (1.04,1.97)	1.35 (1.00,1.92)	0.330
LDL-C (mmol/L)	2.46 (1.90,3.09)	2.33 (1.80,2.95)	***0.001***	2.42 (1.86,3.04)	2.35 (1.82,2.96)	0.136
HDL-C (mmol/L)	0.99 (0.85,1.18)	1.01 (0.87,1.21)	***0.012***	1.00 (0.86,1.20)	1.01 (0.87,1.21)	0.171

PSM, Propensity Score Matching; IQR, Interquartile Range; NIHSS, National Institutes of Health Stroke Scale; TOAST, Trial of Org 10,172 in Acute Stroke Treatment; LAA, large-artery atherosclerosis; CE, cardio embolism; SVO, small-vessel occlusion; ODE, stroke of other determined etiology; UDE, stroke of undetermined etiology; LDL-C, Low-Density Lipoprotein Cholesterol; HDL-C, High-Density Lipoprotein Cholesterol; COPD, Chronic Obstructive Pulmonary Disease; *p* values in bold indicate statistical significance at the *p* < 0.05 level.

### 3.3 Logistic regression exploring independent risk factors for post-stroke pneumonia

The results of the univariate and multivariate logistic regression analyses for PSP risk are presented in Table 2 (before PSM) and Table 3 (after PSM). In the pre-matched cohort, High-intensity atorvastatin therapy appeared as a protective factor in univariate analysis (OR = 0.681, 95% CI: 0.522– −0.892, *p* = 0.005). However, after multivariate adjustment for confounders, it was no longer significantly associated with PSP risk (OR = 1.085, 95% CI: 0.770–−1.541, *p* = 0.644). This neutral association was confirmed in the post-matched multivariate analysis (OR = 1.004, 95% CI: 0.677–1.488, *p* = 0.985).

**Table 2:** Univariate and multivariate logistic regression analysis for post-stroke pneumonia risk before propensity score matching.

Variable	Univariate			Multivariate		
	β	*p* value	OR (95%CI)	β	*p* value	OR (95%CI)
High-intensity atorvastatin therapy group	−0.384	***0.005***	0.681 (0.522–0.892)	0.082	0.644	1.085 (0.770–1.541)
Age (years)	0.051	***<0.001***	1.053 (1.040–1.066)	0.018	***0.023***	1.018 (1.003–1.035)
Smoking history	−0.059	0.665	0.943 (0.720–1.229)	-	-	-
Atrial fibrillation	1.291	***<0.001***	3.635 (2.469–5.249)	0.184	0.508	1.202 (0.690–2.056)
Coronary heart disease	0.571	0.098	1.769 (0.847–3.322)	-	-	-
Consciousness disorder	2.078	***<0.001***	7.992 (5.934–10.730)	0.034	0.881	1.034 (0.660–1.605)
Nasogastric tube	3.248	***<0.001***	25.734 (19.116–34.890)	1.674	***<0.001***	5.333 (3.475–8.200)
Endotracheal intubation/tracheostomy	4.097	***<0.001***	60.133 (33.134–117.106)	2.283	***<0.001***	9.805 (4.782–21.734)
Dysphagia	1.427	***<0.001***	4.168 (3.081–5.598)	0.464	***0.020***	1.591 (1.068–2.345)
Admission NIHSS score	0.166	***<0.001***	1.180 (1.156–1.206)	0.036	***0.013***	1.037 (1.008–1.067)
Stroke location						
Anterior circulation	Ref	Ref	Ref	Ref	Ref	Ref
Anterior & posterior circulation	0.613	***<0.001***	1.847 (1.401–2.429)	0.359	0.051	1.432 (0.997–2.051)
Posterior circulation	−0.467	0.085	0.627 (0.355–1.035)	−0.411	0.232	0.663 (0.325–1.259)
TOAST classification						
LAA	Ref	Ref	Ref	Ref	Ref	Ref
CE	1.516	***<0.001***	4.556 (3.410–6.068)	0.291	0.182	1.338 (0.869–2.045)
SVO	−1.619	***0.006***	0.198 (0.049–0.528)	−0.622	0.302	0.537 (0.129–1.489)
ODE	−18.403	0.976	0.000 (0.000–25131.778)	−14.822	0.970	0.000 (0.000–207.301)
UDE	0.733	***0.035***	2.081 (0.993–3.925)	−0.307	0.558	0.735 (0.248–1.939)
White blood cell count (x10^9^/L)	0.255	***<0.001***	1.291 (1.235–1.350)	0.137	***<0.001***	1.147 (1.086–1.212)
Lymphocyte count (x10^9^/L)	−1.550	***<0.001***	0.212 (0.159–0.281)	−0.811	***<0.001***	0.445 (0.317–0.617)
Albumin (g/L)	−0.127	***<0.001***	0.880 (0.851–0.911)	−0.023	0.282	0.977 (0.937–1.019)
Triglycerides (mmol/L)	−0.993	***<0.001***	0.371 (0.283–0.476)	−0.348	***0.017***	0.706 (0.524–0.927)
HDL-C (mmol/L)	−0.154	0.546	0.858 (0.517–1.399)	-	-	-
LDL-C (mmol/L)	−0.027	0.721	0.974 (0.839–1.125)	-	-	-

PSM, Propensity Score Matching; 95% CI, 95% Confidence Interval; OR, Odds Ratio; NIHSS, National Institutes of Health Stroke Scale; TOAST, Trial of Org 10,172 in Acute Stroke Treatment; LAA, large-artery atherosclerosis; CE, cardio embolism; SVO, small-vessel occlusion; ODE, stroke of other determined etiology; UDE, stroke of undetermined etiology; LDL-C, Low-Density Lipoprotein Cholesterol; HDL-C, High-Density Lipoprotein Cholesterol; *p* values in bold indicate statistical significance at the *p* < 0.05 level.

**Table 3:** Univariate and multivariate logistic regression analysis for post-stroke pneumonia risk after propensity score matching.

Variable	Univariate			Multivariate		
	β	*p* value	OR (95%CI)	β	*p* value	OR (95%CI)
High-intensity atorvastatin therapy group	−0.063	0.691	0.939 (0.687–1.282)	0.004	0.985	1.004 (0.677–1.488)
Age (years)	0.049	***<0.001***	1.051 (1.035–1.066)	0.022	***0.020***	1.022 (1.004–1.042)
Smoking history	0.012	0.944	1.012 (0.729–1.394)	-	-	-
Atrial fibrillation	0.942	***<0.001***	2.565 (1.610–3.964)	−0.014	0.964	0.986 (0.518–1.829)
Coronary heart disease	0.483	0.213	1.621 (0.703–3.275)	-	-	-
Consciousness disorder	1.984	***<0.001***	7.273 (5.128–10.280)	−0.048	0.857	0.953 (0.563–1.593)
Nasogastric tube	3.249	***<0.001***	25.754 (17.982–37.374)	1.929	***<0.001***	6.880 (4.113–11.588)
Endotracheal intubation/tracheostomy	3.952	***<0.001***	52.046 (26.357–112.516)	2.223	***<0.001***	9.234 (4.113–22.744)
Dysphagia	1.342	***<0.001***	3.829 (2.653–5.465)	0.279	0.247	1.321 (0.818–2.104)
Admission NIHSS score	0.159	***<0.001***	1.172 (1.144–1.202)	0.031	0.069	1.031 (0.998–1.067)
Stroke location						
Anterior circulation	ref	ref	ref	ref	ref	ref
Anterior & posterior circulation	0.718	***<0.001***	2.050 (1.477–2.840)	0.456	***0.037***	1.579 (1.025–2.425)
Posterior circulation	−0.246	0.455	0.782 (0.388–1.430)	−0.339	0.433	0.712 (0.288–1.588)
TOAST classification						
LAA	Ref	Ref	Ref	Ref	Ref	Ref
CE	1.407	***<0.001***	4.086 (2.907–5.721)	0.206	0.420	1.228 (0.741–2.015)
SVO	−1.748	***0.015***	0.174 (0.029–0.557)	−0.651	0.379	0.522 (0.083–1.766)
ODE	−12.896	0.975	0.000 (NA-3268870.511)	−14.879	0.979	0.000 (NA-19,409,381,544.001)
UDE	0.570	0.200	1.769 (0.666–3.930)	−0.121	0.849	0.886 (0.231–2.835)
White blood cell count (x10^9^/L)	0.246	***<0.001***	1.279 (1.213–1.350)	0.100	***0.003***	1.105 (1.035–1.179)
Lymphocyte count (x10^9^/L)	−1.471	***<0.001***	0.230 (0.163–0.320)	−0.678	***<0.001***	0.507 (0.340–0.746)
Albumin (g/L)	−0.111	***<0.001***	0.895 (0.859–0.932)	−0.014	0.571	0.986 (0.938–1.035)
Triglycerides (mmol/L)	−0.966	***<0.001***	0.381 (0.276–0.512)	−0.342	***0.050***	0.710 (0.495–0.981)
HDL-C (mmol/L)	−0.150	0.610	0.861 (0.479–1.512)	-	-	-
LDL-C (mmol/L)	−0.037	0.682	0.964 (0.805–1.147)	-	-	-

PSM, propensity score matching; 95% CI, 95% confidence Interval; OR, odds ratio; NIHSS, National Institutes of Health Stroke Scale; TOAST, Trial of Org 10,172 in acute stroke treatment; LAA, large-artery atherosclerosis; CE, cardio embolism; SVO, small-vessel occlusion; ODE, stroke of other determined etiology; UDE, stroke of undetermined etiology. LDL-C, Low-Density Lipoprotein Cholesterol; HDL-C, High-Density Lipoprotein Cholesterol; *p* values in bold indicate statistical significance at the *p* < 0.05 level.

**Figure 3:**
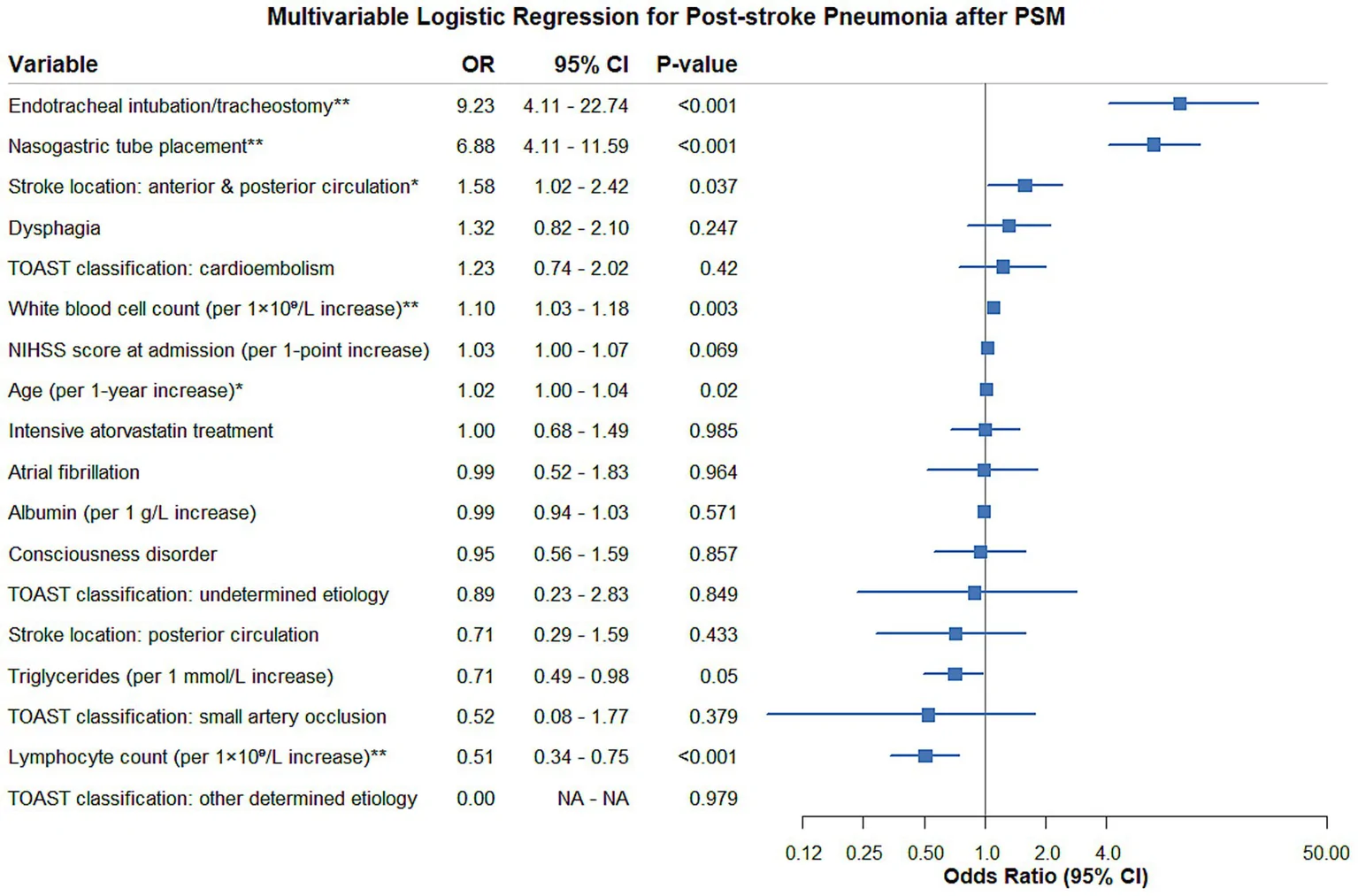
Forest plot of independent risk factors for post-stroke pneumonia after propensity score matching (PSM) (*indicates independent risk factors).

Multivariate models consistently identified several independent risk factors for PSP across both cohorts. The presence of a nasogastric tube, endotracheal intubation/tracheostomy, a higher white blood cell count, and the stroke location (both anterior and posterior circulation stroke) were significantly associated with an increased risk of PSP. Conversely, a higher lymphocyte count and triglyceride level were identified as consistent protective factors (Figure 3).

**Figure 4:**
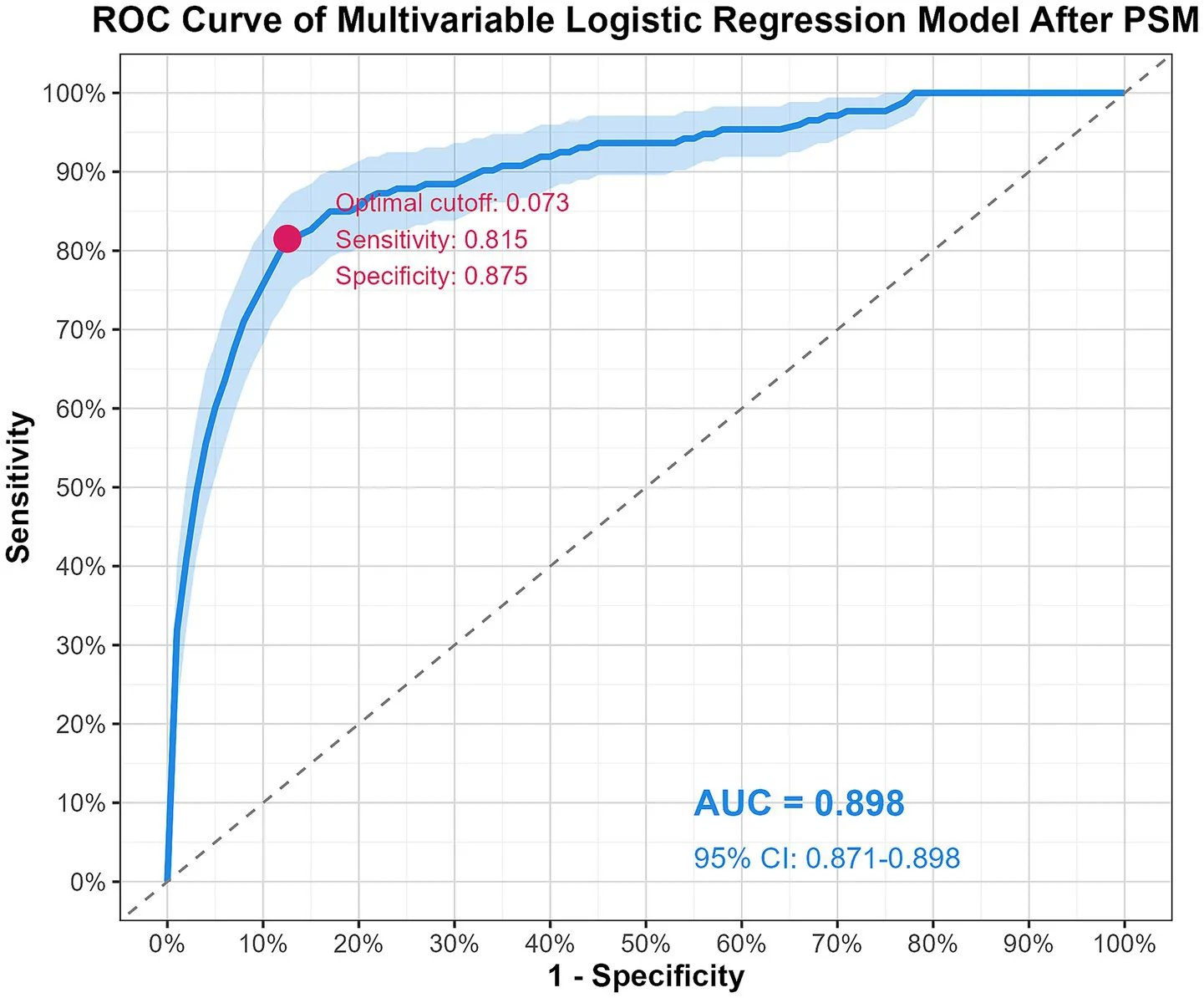
Receiver operating characteristic (ROC) curve of the multivariable logistic regression model after propensity score matching (PSM).

To evaluate the stability and predictive performance of the multivariable logistic regression model, multicollinearity diagnostics, goodness-of-fit testing, and receiver operating characteristic (ROC) curve analysis were performed. The variance inflation factor (VIF) values for all variables were less than 2, indicating no significant multicollinearity. The Hosmer-Lemeshow test results suggested that the model exhibited good fit. ROC curve analysis demonstrated that the area under the curve (AUC) of the model before and after propensity score matching was 0.898 (95% CI: 0.871–0.925), indicating favorable predictive performance (Figure 4).

### 3.4 Impact of different atorvastatin doses on post-stroke pneumonia severity and multidrug-resistant organisms

The analysis of pneumonia severity and MDRO infection rates among the patients who developed PSP is summarized in Table 4. Before PSM, among 243 PSP patients, there were no significant differences between the intensive and standard treatment groups in the CURB-65 score distribution (*p* = 0.089), the proportion of severe pneumonia (*p* = 0.317), or the rate of MDRO infection (*p* = 0.637). After PSM, in the balanced cohort of 173 PSP patients, these findings remained consistent. The severity of pneumonia, as measured by CURB-65 scores and severity categories, was comparable between the two groups (*p* = 0.688 and *p* = 0.863, respectively). Furthermore, the incidence of MDRO infection was not significantly different (9.5% vs. 10.1%, *p* = 0.897).

**Table 4:** Severity and multidrug-resistant organism infection in post-stroke pneumonia patients before and after propensity score matching.

Characteristic	Before PSM			After PSM		
	PSP patients (*n* = 243)			PSP patients (*n* = 173)		
	Intensive treatment (*n* = 144)	Standard treatment (*n* = 99)	*p* value	Intensive treatment (*n* = 84)	Standard treatment (*n* = 89)	*p* value
CURB-65 score, median (IQR)	2 (1–2)	2 (1–2)	0.089	2 (1–2)	2 (1–2)	0.688
PSP severity, *n* (%)			0.317			0.863
Mild	58 (40.3)	31 (31.3)		26 (31.0)	31 (34.8)	
Moderate	59 (41.0)	44 (44.4)		40 (47.6)	40 (44.9)	
Severe	27 (18.8)	24 (24.2)		18 (21.4)	18 (20.2)	
MDRO infection [yes, *n* (%)]	12 (8.3)	10 (10.1)	0.637	8 (9.5)	9 (10.1)	0.897

PSM, propensity score matching; PSP, post-stroke pneumonia; MDRO, multidrug-resistant organism; IQR, interquartile range; *p* values < 0.05 indicate statistical significance.

## 4 Discussion

This study retrospectively analyzed 3,199 patients with acute ischemic stroke (AIS) to systematically evaluate the impact of high-intensity atorvastatin therapy during the acute phase on the risk, severity, and pathogenetic drug resistance of post-stroke pneumonia (PSP). Our primary finding was that, after effectively balancing baseline characteristics between groups using propensity score matching (PSM), there were no statistically significant differences between the high-intensity atorvastatin therapy group and the standard therapy group in terms of the incidence of post-stroke pneumonia, pneumonia severity (measured by the CURB-65 score), or the rate of multidrug-resistant organism (MDRO) infection. Neither before nor after matching did high-intensity atorvastatin therapy significantly increase the risk of post-stroke pneumonia (pre-matching multivariable OR = 1.085, 95% CI: 0.770–1.541, *p* = 0.644; post-matching multivariable OR = 1.004, 95% CI: 0.677–1.488, *p* = 0.985), nor did it exacerbate pneumonia severity or increase the rate of MDRO infection (*p* > 0.05). This negative finding carries important clinical implications, as it unequivocally refutes the potential concern in clinical practice that “intensive statin therapy might increase PSP risk due to excessive immunosuppression.” Our study provides key evidence regarding the safety of statins in the acute phase of AIS and supports clinicians in adhering to guideline-recommended intensive lipid-lowering therapy without the need to reduce dosing intensity due to infection concerns. This finding is consistent with the pleiotropic mechanisms of statins, including immunomodulation, anti-inflammation, and potential antibacterial effects (8, 9), and simultaneously provides an important basis for the safe use of intensive statin therapy in clinical practice.

Post-stroke pneumonia is one of the most common and serious complications of acute stroke, involving multiple pathogenic mechanisms such as aspiration, neurological dysfunction, and stroke-induced immunodepression syndrome (SIDS). Beyond their lipid-lowering effects, statins possess anti-inflammatory and immunomodulatory pleiotropic properties (8, 9), which theoretically may influence post-stroke infection risk. Preclinical studies have shown that simvastatin can attenuate peripheral immunosuppression and reduce susceptibility to pulmonary bacterial infection in mice by inhibiting splenocyte apoptosis and mitigating post-stroke splenic atrophy (25). Atorvastatin has also been demonstrated to alleviate neuroinflammation by modulating the gut microbiota, improving intestinal barrier function, and reducing systemic inflammation (26). Additionally, statins exhibit direct antibacterial activity and the ability to inhibit biofilm formation, particularly against drug-resistant organisms such as methicillin-resistant *Staphylococcus aureus* (MRSA) (27–29).

However, our study did not observe that high-intensity atorvastatin therapy translated into a significant preventive or exacerbating effect on pneumonia in the clinical setting. This result may reflect the complexity of the pathogenic mechanisms underlying post-stroke infection. First, SIDS involves multiple pathways, including sympathetic nervous system activation, hypothalamic–pituitary–adrenal axis excitation, splenic atrophy, peripheral lymphocyte apoptosis, and gut microbiota dysbiosis (7, 30, 31), representing a systemic immune remodeling process. In comparison, the immunomodulatory effects of statins may be relatively modest and insufficient to reverse the complex neuro-immune axis disturbances within the short acute-phase timeframe. Second, animal models typically employ pre-administration of statins or specific pathogen challenges, which differ substantially from the heterogeneity and complexity of secondary infections observed clinically after stroke. Moreover, our study did not include immunological parameters (e.g., CD4+/CD8 + lymphocyte subsets, interleukin-6, interleukin-10, or human leukocyte antigen DR expression), and thus we could not directly verify the impact of statins on post-stroke immune status from an immunoregulatory perspective; this mechanistic inference remains primarily based on previous research findings.

Furthermore, we observed a lower incidence of PSP in the intensive therapy group before PSM, but this difference disappeared after matching to balance baseline variables, suggesting that the original difference may have arisen from baseline imbalance. In clinical practice, patients in the intensive treatment group tended to have better overall condition, lower neurological deficit scores, and fewer invasive procedures—factors that are themselves important determinants of PSP. Without proper confounding control, a “superficial protective effect” could easily emerge. Thus, the results obtained after controlling for bias using both multivariable regression and PSM are more likely to reflect the true effect of the dose itself.

A key finding of this study is that the association between high-intensity atorvastatin therapy and PSP changed markedly before versus after PSM. In the unmatched original analysis, the high-intensity group exhibited a “protective” illusion (PSP incidence: 6.7% vs. 9.5%, OR = 0.681, *p* = 0.005). However, analysis of the pre-matching baseline data (Table 1) revealed that this “protective effect” was largely attributable to severe baseline imbalances between the two groups: clinicians tended to preferentially prescribe high-intensity atorvastatin to patients who were younger, had milder conditions (e.g., lower admission NIHSS scores, lower proportions of atrial fibrillation and coronary heart disease), and underwent fewer invasive procedures (e.g., indwelling nasogastric tube, endotracheal intubation)—factors that are themselves known protective factors or strong predictors of PSP.

Through PSM, we successfully constructed a highly balanced cohort (1,016 pairs) with well-matched baseline characteristics (SMD < 0.1 for all covariates), effectively eliminating the aforementioned selection bias. After matching, there was no longer any statistical association between high-intensity statin therapy and PSP risk (OR = 1.004, 95% CI: 0.677–1.488, *p* = 0.985). This fundamental shift in results precisely demonstrates the necessity and value of implementing PSM, clearly revealing that failure to control for confounding in real-world observational studies can readily produce spurious conclusions of statin “benefit” or “harm.” After rigorously stripping away confounding factors through appropriate statistical methods, our study confirms that the dose of atorvastatin itself is not an independent factor influencing PSP risk. Therefore, the application of PSM substantially enhanced the robustness and credibility of our conclusion—that intensive statin therapy does not increase PSP risk.

Our results are consistent with those of Li et al. (12), who similarly found no significant association between acute-phase statin therapy and post-stroke pneumonia risk. Li et al. speculated that their negative finding might be related to the failure to distinguish between statin types and doses, whereas our study specifically addressed the intensive dose of a particular statin (atorvastatin) and still reached a negative conclusion. It should be noted that our study only included atorvastatin and did not involve other statins such as rosuvastatin or simvastatin; therefore, our conclusions are primarily applicable to atorvastatin and cannot be directly extrapolated to all statins.

On the other hand, our results differ from some studies reporting protective effects of statins. For example, Scheitz et al. found that continued statin therapy in stroke patients receiving intravenous thrombolysis reduced pneumonia risk (13); Song and Kim, in a Korean population-based cohort study, demonstrated long-term benefits of statin therapy in preventing post-stroke pneumonia (15). These discrepancies may be attributed to several factors: differences in study populations (our study excluded patients with prior stroke history or pre-existing statin use, focusing on a “statin-naïve” population), timing and duration of therapy (our study focused on the acute phase, whereas long-term secondary prevention effects may differ), and control of confounding factors. Through PSM and multivariable regression, our study revealed that the intensive therapy group had more favorable baseline characteristics before matching (e.g., younger age, fewer invasive procedures), which could create an illusion of statin benefit in some observational studies. After balancing these confounders, statins themselves showed neither a protective effect nor an increased risk of pneumonia.

Basic research suggests that the immunomodulatory effects of statins may be dose-dependent. Van der Meij et al. (32) found in patients with abdominal aortic aneurysms that statins inhibited vascular wall inflammatory cytokines IL-6 and monocyte chemoattractant protein-1 (MCP-1) in a dose-dependent manner. Ou et al. (16) also demonstrated in septic patients that high-intensity statins improved outcomes more than low-intensity statins. Other studies have suggested that higher-dose statins might be more beneficial in preventing post-stroke pneumonia (15). However, our study did not observe an advantage of intensive-dose atorvastatin in post-stroke pneumonia, suggesting that within the specific time window of acute stroke, immune system dysregulation may not be sufficiently modulated by simply increasing the statin dose to initiate or amplify its protective immunoregulatory pathways.

Our multivariable regression analysis identified independent risk factors for PSP, including indwelling nasogastric tube, endotracheal intubation/tracheostomy, and swallowing dysfunction. These factors are directly related to aspiration risk, representing a core mechanism of post-stroke pneumonia. Concurrently, a lower lymphocyte count was confirmed as an independent risk factor (post-matching OR = 0.507, 95% CI: 0.340–0.746), directly corroborating the “stroke-induced immunosuppression” theory (7, 33, 34), wherein lymphocyte reduction and dysfunction are key to patient susceptibility to infection. Our study also found that higher triglyceride levels were associated with a reduced risk of post-stroke pneumonia (post-matching OR = 0.710, 95% CI: 0.495–0.981). However, this result should be interpreted with caution. On one hand, our study did not include systematic nutritional assessment indicators (e.g., body mass index, albumin), making it difficult to ascertain whether triglycerides simply served as a proxy for nutritional status; on the other hand, this association could also be influenced by residual confounding or reverse causality. Notably, the *p* value for this association was exactly at the borderline of statistical significance (0.050), and the possibility of sample size or chance effects cannot be excluded. Therefore, triglycerides cannot yet be regarded as an independent protective factor, and the underlying mechanisms require further investigation. High-intensity atorvastatin therapy did not significantly modify the risk conferred by these core risk factors, further illustrating its limited role in the pathological cascade of post-stroke pneumonia, while also not increasing pneumonia severity or MDRO infection risk.

In terms of etiology, post-stroke pneumonia is predominantly caused by Gram-negative bacteria, commonly *Pseudomonas aeruginosa*, *Acinetobacter baumannii*, and *Klebsiella pneumoniae*, with a high rate of MDRO infection (35, 36). Although statins have demonstrated *in vitro* and in animal models inhibitory effects against various bacteria, including MRSA and vancomycin-resistant enterococci (27–29), and can reduce bacterial virulence and enhance host clearance (28, 37–39), our study found no significant difference in MDRO infection rates between the intensive and standard therapy groups. This suggests that the antibacterial potential of statins in clinical practice may be limited by multiple factors or may need further validation in specific populations or against specific pathogens. However, given the limited number of MDRO events in our subgroup analysis, this finding should be interpreted with caution and considered hypothesis-generating rather than confirmatory. The small sample size for this specific endpoint precludes definitive conclusions regarding the relationship between intensive atorvastatin therapy and MDRO risk. And our analysis was based on overall infection rates without stratified evaluation of specific bacterial strains and their resistance profiles (e.g., *A. baumannii*, *K. pneumoniae*), so we cannot definitively determine the potential differential effects of statins on various drug-resistant strains. Larger, dedicated studies with adequate statistical power are needed to thoroughly evaluate whether intensive statin therapy influences the etiology or drug-resistance profile of pathogens in PSP.

The strengths of our study include a relatively large sample size with sufficient PSP events, providing adequate statistical power; a focus on comparing “intensive dose versus standard dose” rather than a simple “users versus non-users” contrast, which aligns more closely with current clinical guideline recommendations; the use of PSM to control baseline differences, enhancing result robustness; and the inclusion of infection severity and drug-resistant bacteria as outcome dimensions, broadening the scope of our findings. Nevertheless, several limitations should be acknowledged. First, this was a single-center retrospective cohort study. Despite using PSM and multivariable logistic regression to control for known confounders, we cannot completely rule out selection bias or the influence of unmeasured confounders. For instance, we did not include patients’ nutritional status, standardized swallowing function assessment scales, or dynamic immune function indicators (e.g., lymphocyte subsets, inflammatory cytokine levels), which may have important effects on PSP development. Second, our data were derived from electronic medical records, with potential for missing data or measurement errors for some variables. Third, we did not subdivide the duration of atorvastatin therapy, limiting our ability to explore the potential impact of long-term maintenance therapy. Fourth, the lack of dynamic monitoring data on patients’ immune function (e.g., lymphocyte subsets, inflammatory cytokines) constrained our ability to interpret the negative results from a mechanistic perspective. Fifth, our study only focused on atorvastatin, and the conclusions may not be directly generalizable to other statins. Sixth, the intensive treatment group in this study combined both 40 mg/d and 80 mg/d atorvastatin regimens. While both doses meet the criteria for intensive therapy according to current guidelines, we acknowledge that this aggregation may have obscured a potential dose-dependent immunomodulatory effect. It remains possible that 80 mg/d exerts different immune effects compared to 40 mg/d, and pooling these two regimens into a single group may have diluted the true association. Additionally, we did not include immunological and microbiological parameters (e.g., immune cell subpopulations and pathogen typing), which restricted in-depth analysis of post-stroke immunosuppression mechanisms and drug-resistant bacterial characteristics; the lack of systematic nutritional assessment also affected interpretation of some findings. Therefore, future multicenter, large-sample, prospective randomized controlled trials incorporating dynamic immunological monitoring are warranted to further validate our conclusions and explore the underlying mechanisms.

## 5 Conclusion

In this retrospective propensity score–matched study, high-intensity atorvastatin therapy administered during the acute phase of ischemic stroke was not associated with an increased risk of post-stroke pneumonia, greater pneumonia severity, or a higher incidence of multidrug-resistant organism infection. These findings provide additional evidence regarding the infectious safety of intensive atorvastatin therapy in patients with acute ischemic stroke. Clinical decisions regarding statin intensity should therefore continue to be guided primarily by established cardiovascular and cerebrovascular prevention benefits rather than concerns about post-stroke infectious complications, and clinical decisions should remain guided by the robust evidence for statins in lipid-lowering and cardiovascular secondary prevention (40, 41). Meanwhile, our study confirmed that indwelling nasogastric tube, endotracheal intubation/tracheostomy, swallowing dysfunction, and decreased lymphocyte count are independent risk factors for PSP, suggesting that clinical prevention strategies should focus more on comprehensive management of these modifiable factors. This study complements the evidence on the infectious safety of intensive statin therapy during the acute phase of stroke from a real-world data perspective, providing an important reference for optimizing clinical treatment strategies. Future multicenter prospective studies are warranted to validate these findings and further explore the underlying immunological mechanisms.

## Data Availability

The raw data supporting the conclusions of this article will be made available by the authors, without undue reservation.
