# Correction: Intensive versus standard statin therapy in acute ischemic stroke: a comparative study on the risk of pneumonia and multidrug-resistant bacterial infections

**DOI:** 10.3389/fneur.2026.1946105

**Published:** 2026-08-20

**Authors:** Zhijun Wen, Zhenming Yang, Yirui Huang, Jianhua Cheng

**Affiliations:** 1 Department of Neurology, The First Affiliated Hospital of Wenzhou Medical University, Wenzhou, Zhejiang, China; 2 Department of Clinical Laboratory, Wenzhou Hospital of Integrated Traditional Chinese and Western Medicine, Wenzhou, Zhejiang, China

**Keywords:** atorvastatin, intensive treatment, ischemic stroke, post-stroke pneumonia, risk factors

A correction has been made to the author list in the article. The author order in the published article was erroneously given as: Zhijun Wen, Zhenming Yang, Jianhua Cheng, Yirui Huang. The correct author order should read: Zhijun Wen, Zhenming Yang, Yirui Huang, Jianhua Cheng.

The original version of this article has been updated.

